# The role of pediatricians in the diagnosis and management of IgE-mediated food allergy: a review

**DOI:** 10.3389/fped.2024.1373373

**Published:** 2024-05-30

**Authors:** Ruchi S. Gupta, Ellen Epstein, Robert A. Wood

**Affiliations:** ^1^ Institute for Public Health and Medicine, Center for Food Allergy & Asthma, Northwestern University, Chicago, IL, United States; ^2^ Novartis Pharmaceuticals Corporation, East Hanover, NJ, United States; ^3^ Department of Pediatrics, Johns Hopkins University School of Medicine, Baltimore, MD, United States

**Keywords:** diagnostic, referral, pediatric clinics, IgE, food allergy

## Abstract

**Importance:**

Food allergy can often cause a significant burden on patients, families, and healthcare systems. The complexity of food allergy management requires a multidisciplinary approach involving different types of healthcare providers, including allergists, dieticians, psychologists, nurses, family practitioners and, of particular relevance for this article, pediatric primary caretakers. Pediatricians may be the first-line healthcare providers for food allergy: strategies for management and guideline adherence have been highlighted.

**Observations:**

This review article summarizes the up-to-date recommendations on the role of pediatricians in the diagnosis, management, and prevention of IgE-mediated food allergy. Early introduction of allergenic foods like peanut is known to be of importance to reduce the development of peanut allergy in infants, and pediatricians are essential for educating and supporting parents in this decision. In scenarios of limited allergist availability, as is often the case among rural, Medicaid and minority populations, pediatricians can assist in the evaluation and management of food allergy, and provide action plans, education and counselling for patients and families.

**Conclusions and relevance:**

Pediatric primary caretakers play a key role in the diagnosis, management, and prevention of IgE-mediated food allergy. As more diagnostic tools and therapies in food allergy become available, the need for a multidisciplinary team is paramount to optimize patient care.

## 1 Introduction

Food allergy, an immune-mediated adverse reaction to food, is a potentially life-threatening condition associated with a significant economic burden and impact on patients' and caregivers' quality of life (1–6). Immune-mediated food allergies include immunoglobulin E (IgE)-mediated food allergies and mixed-IgE and non-IgE-mediated allergy (7). Most food allergy is IgE-mediated; types of non-IgE-mediated allergy include eosinophilic gastrointestinal diseases and food protein-induced enterocolitis syndrome (FPIES) (6, 7). Since food intolerance is also common and can have a similar clinical presentation to food allergy, a key challenge for healthcare professionals is to distinguish immune-mediated food allergy from food intolerance (7).

With prevalence rates on the rise, IgE-mediated food allergy is estimated to affect up to ∼8% of children and 10.8% of adults in the United States (US) (8, 9). Self-reported prevalence estimates of food allergy are even higher, with >11% of children perceived as food-allergic by their caregivers and nearly 19% of adults believing they have a food allergy (8, 9). In patients with IgE-mediated food allergies, approximately 40% of children and 45% of adults are allergic to multiple foods (8, 9). These discrepancies highlight the need for improved diagnosis, confirmatory testing, preventative strategies and early management of food allergy in order to prevent unnecessary food avoidance and impact on quality of life (9).

Patients may receive support from a variety of different healthcare providers in different scenarios in the management of their food allergies, including primary and secondary care providers, family practitioners, and a wider multidisciplinary team. This review will focus on pediatricians, who are often the first-line healthcare providers (depending on the individual healthcare system and the healthcare access of the patient); they play a vital role in ensuring appropriate preventative measures through early introduction and the diagnosis and management of food allergy (10). However, knowledge gaps exist and variations in adherence to guidelines across pediatric clinics have been highlighted (11–13). This article aims to provide a comprehensive review of the diagnosis, management, and prevention of food allergy by pediatricians and the wider multidisciplinary team, with the aim of improving accurate diagnosis and optimizing evidence-based pediatric food allergy patient care.

## 2 Diagnosis of food allergy

### 2.1 Medical history, comorbidities, and family history

In the US, practice recommendations for the diagnosis and management of food allergy were established in the 2010 National Institute of Allergy and Infectious Diseases (*N*IAID) guidelines (5, 6) and are informed by recent guidelines such as practice parameters developed by the American Academy of Allergy, Asthma & Immunology (AAAAI); the American College of Allergy, Asthma & Immunology (ACAAI); and the Joint Council of Allergy, Asthma & Immunology (JCAAI) (14) and the Global Allergy and Asthma European Network (GA^2^LEN) guideline (15). For patients with suspected food allergy, a thorough medical history is the first step in making an accurate diagnosis. Such a medical history should include documenting all foods eaten prior to the reaction, severity and duration of symptoms, time elapsed between ingestion of the food and the onset of symptoms, and response to medications (5, 6). An IgE-mediated reaction is suspected when the onset of symptoms is less than 2 h after ingestion (10). Skin manifestations and oral symptoms are usually but not always the first to appear in an IgE-mediated reaction (10). The type of allergen should also be considered when making the diagnosis: reactions to peanuts, tree nuts and shellfish are almost always IgE-mediated, whereas other foods such as milk, wheat and soy could be IgE-mediated, non-IgE-mediated, or mixed (7, 14). It is important to differentiate between IgE- and non-IgE-mediated reactions such as FPIES and food protein-induced allergic proctocolitis (FPIAP), for which there are no biomarkers and diagnosis relies upon recognition of symptom constellation (16, 17). Management of these conditions generally involves removal of the trigger food, which typically resolves FPIES and FPIAP (17), and reintroduction at a later stage under physician supervision for FPIES, as per international consensus guidelines (18).

Food allergic children often present with comorbidities such as atopic dermatitis, asthma and/or allergic rhinitis, as these are linked by the “atopic march” (19, 20). The presence of comorbidities may also affect symptom severity and treatment response, with uncontrolled asthma as a risk factor for severe anaphylaxis (6), although fatalities remain rare (approximately 0.03–0.3 deaths per million person years in the general US population) (21). Food allergy testing should not be a standard part of evaluation of children with respiratory allergies or atopic dermatitis, but a subset of patients with severe, treatment-refractory atopic dermatitis may benefit from food allergy testing if there is a suspicion that allergen ingestion is linked to flares (6, 22).

Atopy in parents can increase the odds of atopic conditions in children, particularly atopic dermatitis (23, 24) but also food allergy (25). However, screening of siblings should not be routinely performed as sibling history is not a major risk factor for developing food allergy (22).

Pollen Food Allergy Syndrome (PFAS), sometimes known as oral allergy syndrome, is an IgE- dependent allergic reaction directed toward fruits, vegetables, and nuts linked to allergic sensitization to pollen, the most frequent symptoms of which is contact urticaria of the oropharyngeal sites (26). Other symptoms of PFAS may include tightness in the throat, difficulty swallowing, dysphonia, nausea, nasal itching, and itching of the ear (27). PFAS complicates the diagnosis and management of food allergies by leading to uncertainty around potential severity of future reactions and indications for prescribing epinephrine, as well as the extent of necessary dietary avoidance (28). The main pan-allergens of relevance for PFAS include three protein families: profilins, pathogenesis-related protein type 10 (PR-10), and nonspecific lipid transfer proteins (LTPs) (26). Bet v 1 (birch) is one of the most studied PR10 proteins (27), and sensitization to Bet v 1 has been shown to be cross-reactive to multiple fruits, vegetables, and nuts e.g., Mal d 1 in apple, Api g 1 in celery, Ara h 8 in peanuts, and Cor a 1 in hazelnuts (29).

### 2.2 Specific IgE and skin-prick testing

Medical history alone is not sufficient to diagnose food allergy, and it should be supplemented by testing including allergen-specific IgE (sIgE) and/or skin prick testing (SPT) (14). Both SPT and sIgE tests are highly sensitive, but non-specific. Therefore, the use of these tests may lead to false positive results (14, 22). These tests should generally only be undertaken when the patient presents with a history of reaction to a given food (14, 22). If a patient has a clear history of eating peanuts and having a reaction consistent with a food allergic reaction (hives, vomiting, trouble breathing, etc) ordering a specific IgE to peanut is the best next step. Do not order testing on any food that is currently tolerated in their diet and do not order panels. If the sIgE is negative it is a good indication that they do not have that IgE-mediated food allergy. If it is positive, it is important to provide counselling, epinephrine autoinjector, and referral to an allergist. A recent expert consensus for the diagnosis of peanut allergy suggested that diagnostic testing for peanut allergy should be utilized in patients with a moderate-to-high pre-test probability of peanut allergy, depending on patient/caregiver preference and prior to oral food challenge (OFC) (22). To avoid misdiagnosis and unnecessary dietary restrictions that can impact nutrition and quality of life, the expert panel discouraged testing in those with a low pre-test probability (22). Food allergen panel testing or the addition of sIgE testing for foods other than peanut were not recommended, due to their poor positive predictive value, which could contribute to misdiagnosis (22).

SPT is widely used by allergists as a means to detect the presence of sIgE bound to mast cells in the skin (10, 30). A positive test is considered as a wheal diameter of ≥3 mm (30), although this is variable depending on the population and food studied (7). Dermatographism, severe atopic dermatitis and use of antihistamine medications are considered contraindications for SPT testing (10). In general, sIgE levels will be most readily available for pediatricians and may be more likely to correlate with current symptoms (14), although it is important to remember that they cannot predict the severity of future reactions which vary among different patient populations (14, 22). Recent data suggest that, although the utilization of confirmatory SPT and sIgE testing is on the rise, over a third of children with convincing food allergy do not receive a physician diagnosis (31).

### 2.3 Oral food challenge (OFC)

In some cases, an OFC may be needed to definitively diagnose food allergy (22). OFCs are the best available tests to evaluate the presence of allergic reactivity to foods (32). OFCs may be needed if SPT and/or sIgE results do not correlate with reaction history, or to determine if a specific food allergy has been outgrown (32).

Due to the risk of false positive results with SPT and/or sIgE testing, OFCs can play a vital role in minimizing misdiagnosis (33). However, they must be performed by an experienced allergist, require intensive resources and can cause anaphylaxis (33, 34). Factors that can increase the risk of severe reactions during an OFC include type of allergen (peanut, tree nuts, fish, shellfish and milk are more commonly implicated in fatal and near-fatal anaphylaxis), comorbid uncontrolled asthma, delayed use of epinephrine, and upright posture during the assessment (32). The patient should also be in good health on the day of the OFC; it is not recommended to perform the test in conditions of concurrent illness, poorly controlled asthma, atopic dermatitis or allergic rhinitis, unstable cardiovascular disease, pregnancy or beta-blocker therapy (32). As such, the individual benefits and risks to the patient should be evaluated in a shared decision-making process to determine the value of performing an OFC on a case-by-case basis (32). There is a consensus that better diagnostic tests are needed to minimize the need for OFCs (33–35).

### 2.4 Other diagnostic tests

Component testing (molecular diagnosis) allows the identification of sIgE for specific proteins, or components, which are available for several allergens including peanut, milk, egg, and several tree nuts (7, 36). Ara h 2 is an example of component testing and is approved for the diagnosis of peanut allergy by the FDA (22, 37). Compared with whole peanut SPT and sIgE tests, Ara h 2 testing is cost-effective and has increased specificity for diagnosis (22). It is important to recognize that component testing is not necessarily needed and that the use of “reflex testing” (testing for multiple allergens irrelevant of reaction history), should be strongly discouraged (22).

Novel techniques such as bead-based epitope assays, basophil activation tests and mast cell tests are also being investigated for use in clinical practice and may help to risk stratify patients in the future, as well as to predict response to immunomodulatory therapy (33, 34). Furthermore, progress in emerging component testing such as Bet v 1 testing or Cor a 9 and Cor a 14 tests may enhance the specificity of testing (27, 38).

### 2.5 Digital technologies

Digital technologies including clinical decision support systems present a promising new avenue for the enhanced detection and management of food allergy in primary care (39). Tools such as the automated allergy management support system (40) and the food allergy support tool (41) were developed to support the detection and management of IgE-mediated food allergy, respectively, in the primary care setting. Further efforts are needed to ensure compatibility with existing software systems and usability in clinical practice in order to facilitate the implementation of such digital support tools in improving patient care (39).

### 2.6 Emergency department

The incidence of emergency department (ED) visits for food allergy is increasing, and is estimated to be between 3%–4% for adults and ∼8% for children (42). While the confirmatory diagnosis of food allergy falls out of the realm of acute care, patients suspected of having a food allergy can be identified based on patient history and clinical presentation (42). It is essential that these patients are discharged with an epinephrine prescription and appropriate patient counselling regarding self-administration and food avoidance (42). The NIAID guidelines recommend a follow-up appointment with a primary care physician, with consideration for referral to an allergist (6).

### 2.7 Barriers to diagnosis

**Figure 1:**
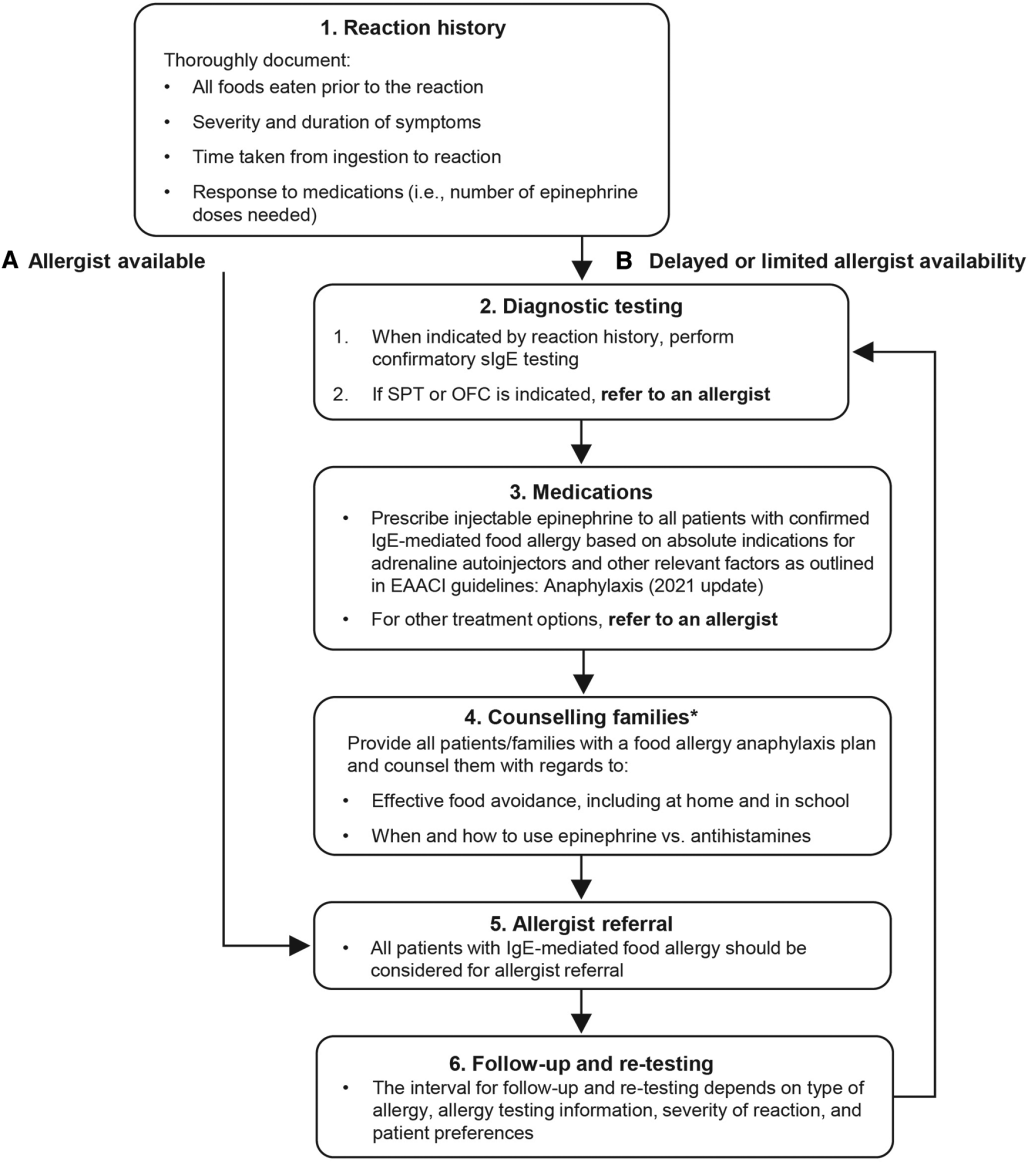
Diagnosis and management of IgE-mediated food allergy by pediatricians, in scenarios of good allergist availability (**A**) or delayed/limited allergist availability (**B**) *A template and guidance for completing the food allergy anaphylaxis plan is available from the American academy of pediatrics. (44).

A key barrier to food allergy diagnosis is access to an allergist in certain groups, for example in Medicaid and minority populations (43). Furthermore, a 2014 study indicated that pediatrician confidence in ordering sIgE tests is variable, with only 34.7% adhering to guidelines for appropriate use of diagnostic tests (12). Although the rate of referral to an allergist was 67.3%, there was a significant delay to referral in over half of all patients (12). In cases of good allergist availability, it may be possible to directly refer patients to an allergist based on their reaction history, thus avoiding duplication of testing (Figure 1A). However, the role of the pediatrician may need to be expanded in incidences of delayed or limited access to an allergist (Figure 1B).

### 2.8 When should an allergist be consulted?

Food allergy significantly impairs quality of life for children with food allergy and their caregivers (1) and leads to increased healthcare resource utilization and costs (45) and substantial population-level psychosocial burden (46). Timely referral to an allergist is of vital importance to reduce the effects of delayed diagnosis on patients. Delay in referral to an allergist can contribute to poor patient experience and increased healthcare resource utilization (47) as well as to the impact of food allergies on patients and caregivers (1). Consultation, and regular follow-up with an allergist should be considered in all children with a convincing diagnosis of IgE-mediated food allergy. Consider the following factors, in particular, for expedited referral:

• Medical history is indicative of anaphylaxis (5, 48)
• Specialized testing such as skin testing for certain food allergens is needed and cannot be accessed from primary care (49)
• OFC testing is needed (48)
• Patient requires repeat instructions on how and when to use injectable epinephrine, especially in those patients with limited adherence and risk-taking behavior (5)
• Food allergy is leading to nutritional concerns (7)
• Shared decision-making is needed regarding treatment options (50)

### 2.9 Follow-up and re-testing

Following the diagnosis of food allergy, yearly follow-up is recommended with an allergist; this may be more frequent in younger patients and those with risk factors such as nutritional concerns, poorly-controlled asthma, risk-taking behavior, frequent accidental ingestions and/or history of severe reactions (6, 7, 14, 37). Frequency of follow-up is also influenced by comorbidities (e.g., asthma, allergic rhinitis, and atopic dermatitis), availability of allergist services and the type/number of food allergies (37).

The interval for periodic re-testing is allergen-dependent (6). Certain allergens (e.g., milk and egg) are outgrown earlier and more frequently than others (e.g., peanut and tree nuts): annual testing is often utilized for the former, with extended intervals of up to 2–3 years for the latter (6). Re-testing may not be necessary in cases of a recent allergic reaction to the food (6). Long-term food allergy management requires shared decision-making based on the type of allergy, allergy testing information, severity of reaction, and patient preferences (50).

## 3 Management options

### 3.1 Avoidance

When food allergy is confirmed, the current mainstay of long-term management remains strict food avoidance; however, accidental exposure is common. Data from the Food Allergy Research and Education (FARE) registry demonstrated that on average, nearly two-thirds of adults and nearly half of children experienced 1 or more food-related allergic reactions per year (51). Data suggest that severe reactions and ED visits are more common in racial minorities and patients from lower income households (52).

### 3.2 Acute treatment: injectable epinephrine

The first-line acute treatment for food-induced anaphylaxis is injectable epinephrine (53). As noted in the NIAID-Sponsored Expert Panel report, all other treatments have a delayed onset of action and repeat epinephrine dosing remains first-line therapy over adjunctive treatments if there is progression of symptoms or if the response to the initial dose of epinephrine is suboptimal (5). Despite its utility in preventing fatal anaphylaxis, there is evidence that epinephrine is still under-prescribed by healthcare providers and under-used by patients (53). Furthermore, hesitancy to use epinephrine when needed among patients and caregivers can also result in undertreatment of allergic reactions (54). A key role of the pediatrician is to effectively counsel patients with regards to when and how to use epinephrine compared with antihistamines (55). Around 2 in 3 children with physician-confirmed food allergy reported a current epinephrine prescription (19). Notably, the likelihood to carry injectable epinephrine is reduced in children from lower income households (52).

Potentially life-threatening reactions can occur anywhere, and since children spend a large proportion of their time at school, it is also crucial to optimize the management of food allergy in the school setting (56–58). Not only do clinicians play a key role in supporting individual patients to manage their food allergy at school, but they have an opportunity through education and advocacy to help optimize school policies and strategies for managing food allergy on a wider scale (56). In particular, the reduced availability of epinephrine in schools of lower socioeconomic status should be addressed (59).

### 3.3 Oral immunotherapy

Guidelines that include oral immunotherapy for the treatment of food allergy have been provided by the European Academy of Allergy and Clinical Immunology since 2018 (60), and in January 2020, peanut oral immunotherapy (POIT) was approved by the FDA in patients aged 4–17 years (61). POIT is administered by gradually increasing doses of allergen, and was shown to be effective at inducing desensitization (62) and improving quality of life (63). Despite this, uptake of POIT in the community appears to be limited, with one study indicating that only 10% of eligible patients and caregivers chose to pursue therapy, due to time commitment and adverse effects (64). Furthermore, access to POIT is limited by the availability of centers with trained staff, particularly in clinical practice outside of academic settings (65). Of the 78 responders to a 15-question survey of 780 randomly selected members of the American Academy of Allergy, Asthma & Immunology, 50% were offering POIT at their practice (65), while another survey with responses from 129 private practices among members of the OIT Advisors group, found that 97.5% had treated at least one patient (66). Adverse effects with POIT can vary from mild gastrointestinal symptoms and skin rash/itching, to severe reactions such as eosinophilic esophagitis (EoE) and anaphylaxis (62). Shared decision-making must be prioritized in allergist consultations around POIT and decision aids have been developed to support this process (67).

### 3.4 Omalizumab for the treatment of food allergy

In February, 2024, omalizumab was approved by the FDA for the treatment of food allergy (68). This monoclonal antibody that specifically blocks the binding of IgE to mast cells and basophils has been approved for the treatment of allergic asthma since 2003, as well as for the treatment of chronic urticaria and chronic rhinosinusitis with nasal polyps. Numerous prior studies have suggested that it could be efficacious for the treatment of food allergy, and this impression was confirmed by the OUtMATCH trial, which demonstrated a high degree of efficacy for patients with multiple food allergies (69–77). Omalizumab is currently approved for the prevention of reactions to small, accidental exposures in all patients with food allergy age 1 year and above.

### 3.5 Investigational treatments

The treatment landscape for food allergy is rapidly evolving; therapeutic strategies under investigation for allergen immunotherapy include subcutaneous immunotherapy (SCIT), sublingual immunotherapy (SLIT), and epicutaneous immunotherapy (EPIT) (78). Several biologics and small molecules in addition to omalizumab, including BTK inhibitor (acalabrutinib and remibrutinib), anti-IL-33 antibody (etokimab), and anti-TSLP antibody (tezepelumab) are being studied to target different pathways in the allergic response (77, 79–81). Clinical trials are also exploring the combination of treatments, for example anti-IgE in combination with OIT, to improve the safety and time to reach maintenance dose (69).

### 3.6 Early introduction of allergen

The NIAID guidelines for the diagnosis and management of food allergy were updated in 2017 with an addendum recommending early introduction of peanut in at-risk children, following results from the Learning Early About Peanut Allergy (LEAP) clinical trial (82). At the 4- and 6-month visits, it is important to discuss peanut product introduction. New guidelines suggest all infants should be encouraged to start peanut products as soon as they are ready for solids after the introduction of initial foods like fruits or vegetables. If the infant has severe eczema or if the parents are apprehensive, a specific IgE to peanut or referral to an allergist can be obtained. If this is positive, it is critical to get them in to see an allergist immediately. New data show the longer the delay in introducing peanut products past six months in high-risk infants, the more likely they will have developed a peanut allergy. Early introduction of other allergenic foods might also have benefit. For example, there is some evidence to support early introduction of egg, which is associated with a lower risk of egg allergy, depending on the nature and dose of egg protein exposure (83, 84). Other allergenic foods also show promise through the Enquiring About Tolerance (EAT) study (85) and are currently being studied in the ongoing CANDO study (NCT05258656). The US Department of Agriculture and Department of Health and Human Services’ Dietary Guidelines for Americans recommend introducing potentially allergenic foods (such as peanuts, egg, cow milk products, tree nuts, wheat, crustacean shellfish, fish, and soy) when other complementary foods are introduced to an infant's diet at 4–6 months (86).

Early introduction of food is considered a preference-sensitive clinical scenario and care should be taken to help parents consider their options (50). In order to enable timely introduction, there may be a role for SPT or sIgE testing in infants with moderate to severe eczema or other food allergies, or when caregivers are highly anxious about introducing peanut into their child's diet; however, it is imperative that these results are interpreted appropriately since a positive result may not be diagnostic of food (22).

### 3.7 Multidisciplinary approach

**Figure 2:**
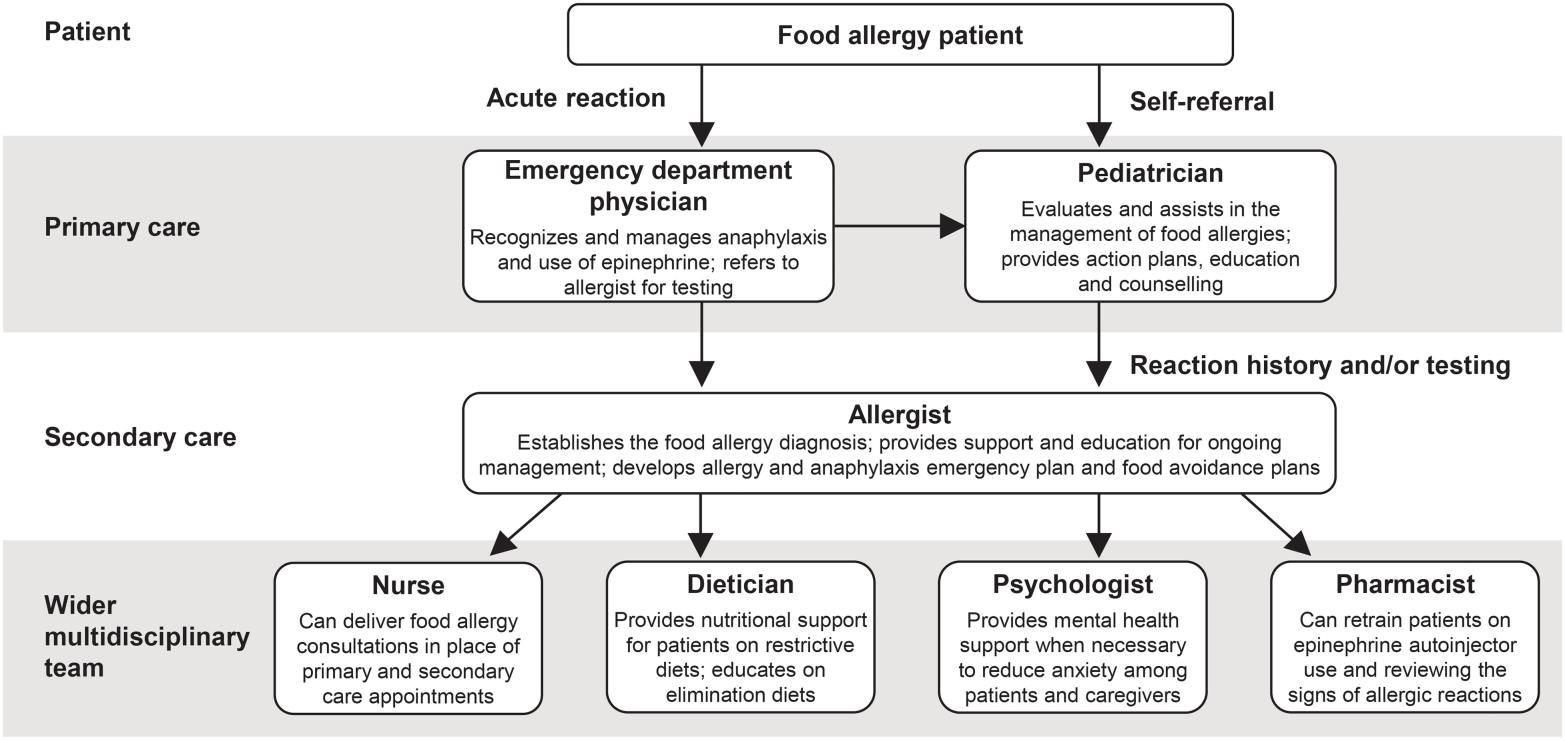
Multidisciplinary management of food allergy.

Pediatricians and allergists are central to the effective multidisciplinary management of food allergies. Furthermore, there may be scope for other members of the multidisciplinary team to play a larger role in patient care (Figure 2) to aid pediatricians and allergists in supporting their patients with food allergy treatment:

• A large feasibility trial demonstrated the potential of nurse-led allergy clinics in delivering remote and face-to-face consultations with patients to ease the burden on primary care services (87)
• Dieticians provide crucial nutritional and dietary support to patients on restrictive diets, as well as education on allergen avoidance, and dietician-led services also have potential to reduce primary and secondary care appointments (88)
• Patients with multiple food allergies may benefit most from nutritional counselling (37) and it is recommended by the NIAID for all food allergy patients (6)
• Community pharmacists are able to work collaboratively with pediatricians and allergists to provide ongoing assistance by retraining patients on the use of epinephrine autoinjectors and reviewing the signs of an allergic reaction (89)
• Food allergies can lead to significant psychosocial impact and mental health support may be beneficial to reduce anxiety among patients and caregivers (90)

## 4 Conclusions

Food allergy leads to significant burden on patients, families, and healthcare systems. Pediatricians play a key role in the prevention, diagnosis, and management of IgE-mediated food allergy, especially in scenarios of limited allergist availability, as is often the case among Medicaid and minority populations. However, allergists should be consulted for all children with food allergy and timely referral is key. As more diagnostic tools and therapies in food allergy become available, the need for a multidisciplinary team is paramount to optimize patient care. A multidisciplinary team comprising nurses, dieticians, and pharmacists, along with paediatricians and allergists, is crucial for accurate diagnosis and appropriate management of food allergy in order to substantially ease the burden on patients, families, and healthcare systems and so that patients can get new treatments as they become available.

### 4.1 Useful links and resources

• Guidelines for the Diagnosis and Management of Food Allergy in the United States: Summary of the NIAID-sponsored Expert Panel Report (6)
• Addendum Guidelines for the Prevention of Peanut Allergy in the United States: Summary for Clinicians (82)
• iREACH training materials: Early Peanut Product Introduction Tools for Pediatric Clinicians (91)
• AAP Allergy and Anaphylaxis Emergency Plan Template (44)
• EAACI Molecular Allergology User Guide 2.0 (92)
